# Nutritional Counseling for Head and Neck Cancer Patients Undergoing (Chemo) Radiotherapy—A Prospective Randomized Trial

**DOI:** 10.3389/fnut.2019.00022

**Published:** 2019-03-18

**Authors:** Helena Orell, Ursula Schwab, Kauko Saarilahti, Pia Österlund, Paula Ravasco, Antti Mäkitie

**Affiliations:** ^1^Unit of Clinical Nutrition Therapy, Helsinki University Hospital, Helsinki, Finland; ^2^Institute of Public Health and Clinical Nutrition, Faculty of Health Sciences, University of Eastern Finland, Kuopio, Finland; ^3^Internal Medicine, Institute of Clinical Medicine, Kuopio University Hospital, Kuopio, Finland; ^4^Department of Oncology, Helsinki University Hospital and University of Helsinki, Helsinki, Finland; ^5^University Hospital of Santa Maria, University of Lisbon, Lisbon, Portugal; ^6^Centre for Interdisciplinary Research in Health (CIIS), Universidade Católica Portuguesa, Lisbon, Portugal; ^7^Department of Otorhinolaryngology-Head and Neck Surgery, Helsinki University Hospital and University of Helsinki, Helsinki, Finland; ^8^Research Programme in Systems Oncology, Faculty of Medicine, University of Helsinki, Helsinki, Finland; ^9^Division of Ear, Nose and Throat Diseases, Department of Clinical Sciences, Intervention and Technology, Karolinska Institutet and Karolinska University Hospital, Stockholm, Sweden

**Keywords:** nutritional status, handgrip strength, nutritional intervention, survival, weight loss

## Abstract

**Background:** Locally advanced head and neck cancer is managed either by combined surgery and (chemo) radiotherapy or definitive (chemo) radiotherapy, which may deteriorate nutritional status. Previous data have shown that intensive nutritional intervention by a dietician reduces radiation-induced adverse events including weight loss.

**Objective:** To determine if on-demand nutritional counseling (ODC, control group) would be as efficacious as intensive nutritional counseling (INC, experimental group) in patients undergoing (chemo) radiotherapy.

**Methods:** Fifty-eight patients were randomly assigned to receive INC (*n* = 26) or ODC (*n* = 32). Outcome measures were nutritional status (PG-SGA), weight loss, handgrip strength (HGS), body composition, and survival.

**Results:** Weight loss and impaired nutritional parameters during oncological treatment were seen equally in both groups (NS). Leaner patients at baseline maintained their weight, while overweight patients lost both weight and handgrip strength during treatment. Disease-free survival (DFS) (median = 43 months) was not affected by weight loss during treatment. Lower baseline HGS and malnutrition were associated with worse DFS (low vs. normal HGS: 15 vs. 42 months; *p* = 0.05 and malnutrition vs. good nutrition status: 17 vs. 42 months; *p* = 0.014, respectively). Survival according to low vs. normal HGS in the INC group was 4 vs. 44 months (*p* = 0.007) and in the ODC group 28 vs. 40 months (*p* = 0.944). According to malnutrition vs. good nutritional status in the INC group, DFS was 21 vs. 43 months (*p* = 0.025) and in the ODC group 15 vs. 41 months (*p* = 0.03).

**Conclusions:** As for our primary endpoint, individualized on-demand nutritional counseling was as efficacious as intensive counseling in preventing deterioration of nutritional status and incidence of malnutrition during (chemo) radiotherapy. This should be verified with larger number of patients. Additional findings were that overweight patients had more severe weight loss, but not poorer survival. Low HGS and malnutrition at baseline were associated with poor survival.

**Clinical Trial Registration:** www.ClinicalTrials.gov, identifier NCT02159508.

## Introduction

Adjuvant chemo (radiotherapy) for locally advanced head and neck squamous cell cancer (HNSCC) results in better survival than radiotherapy alone but causes worse oral and systemic symptoms and decreased food intake and thereby causes weight loss and adverse outcomes (1). Nutritional treatment has an essential role in the management of HNSCC to prevent both disease and treatment-related weight loss (2). In addition, malnutrition prior to diagnosis and during treatment predicts poorer survival in HNSCC (3, 4).

Weight loss is a major predictive marker for impaired response to cancer treatment and poor survival (3, 5, 6). Most patients with locally advanced HNSCC report weight loss already before diagnosis, which tends to persist during anti-neoplastic treatments, mainly due to acute adverse effects of chemoradiotherapy (6). The reported incidence of critical weight is around 17–28% before treatment and 50–80% at the end of treatment, depending on the tumor location, stage, and treatment modality (7–11). A recent study on nasopharyngeal cancer showed that 20% of patients lost more than 10% of weight during chemoradiotherapy (12). There are a few studies in patients with head and neck cancer referred to adjuvant anti-neoplastic treatments demonstrating that early nutritional intervention stabilizes nutritional status and improves nutritional intake (2, 5, 13–15), furthermore the American Dietetic Association Medical Nutrition Therapy Protocol has been found to be effective in physiological and clinically relevant outcomes in head and neck cancer patients undergoing radiotherapy (2, 13, 14, 16).

In head and neck cancer, benefits on nutritional status, nutritional intake, functional status, symptoms, and quality of life have been demonstrated when individualized counseling is performed vs. no counseling or vs. general nutrition advice given by a nurse (5, 17–19). More importantly, a randomized controlled trial in colorectal cancer patients showed improved survival in patients who received individualized nutritional counseling when compared with the group that received standard of care (20).

The potential difference in the effect of individualized intensive nutritional counseling given by a dietician vs. on-demand individualized counseling has not been previously explored. This is a topical issue due to the need to provide dietetic services more efficiently and with limited resources as expected. The primary endpoint measure of this study was the efficacy of nutritional intervention on nutritional status. We established as secondary endpoints the efficacy of intensive nutritional counseling on body composition, handgrip strength, treatment-related adverse events, and survival. The results will be useful for the development of a nutritional treatment protocol at our Department, potentially adaptable by other departments.

## Materials and Methods

### Trial Design

This open labeled, parallel-group, exploratory randomized trial was conducted at the Helsinki University Hospital, Helsinki, Finland. The study design followed the guidelines laid down in the Declaration of Helsinki and all procedures involving human patients were approved by the institutional Research Ethics Committee. Written informed consent was obtained from all patients.

### Patients

Inclusion criteria were: patients with locally advanced (Stage III-IV) squamous cell carcinoma of the oral cavity, oropharynx, hypopharynx, nasopharynx, or larynx, referred for a curative treatment with combined surgery and adjuvant (chemo) radiotherapy, or definitive (chemo) radiotherapy, who were 18–80 years old and gave their written informed consent. Exclusion criteria included: renal function impairment (serum creatinine >1.5 times upper limit of normal [ULN]), liver failure (serum bilirubin >1.5 times ULN), heart failure, *cor pulmonale*, COPD or cognitive impairment. Patients were also excluded if they had had a previous cancer in any location, or if they were recommended for palliative treatment with no curative therapeutic options.

This study was conducted between November 2007 and December 2009. Eligible patients according to the inclusion criteria were randomly assigned to one of the two study groups: intensive nutritional counseling (INC, experimental group) or on-demand counseling (ODC, control group); the groups differed in the number of nutrition consultations during treatment; INC consisted of protocol counseling given by a dietician at baseline, on the 2nd and 4th week of treatment, and at the end of chemoradiotherapy. In the ODC group patients received baseline nutritional counseling, that consisted of one dietetic consultation before chemoradiotherapy. During chemoradiotherapy ODC patients received counseling only on demand. In the ODC group, the criteria for physicians to request for nutritional intervention were any concerns regarding intake (typically weight loss >5%, or any symptoms referring to significant decrease in nutritional intake). There were three staff physicians involved in this trial who were fully aware of the criteria for referring patients for a dietician consultation.

Randomization was performed by the minimization procedure (21) with the Minim Program® (http://www-users.york.ac.uk~mb55/guide/randsery.htm). The allocation was done according to the following criteria: (1) Stage I-II vs. Stage III-IV; (2) age < 65 vs. ≥65 year; (3) Body Mass Index (BMI) < 20 vs. ≥20 kg/m^2^, and (4) tumor location (oral cavity-oropharynx-tonsils vs. hypopharynx-larynx vs. nasopharynx). The allocation ratio was 1:1. Randomization into the INC and ODC groups was performed after the cancer diagnosis had been established and oncological treatment approach for each patient had been discussed at the multidisciplinary tumor board meeting.

Management decisions were based on the Finnish national guidelines for the treatment of HNSCC and were dependent on the tumor location and stage and on patient's general health status. Patients were recruited at their first outpatient visit at the Department of Otorhinolaryngology—Head and Neck Surgery of Helsinki University Hospital after the Multidisciplinary Tumor Board Meeting.

### Surgery and Oncologic Treatment

Tumor resection in the oral cavity and/or oropharynx was typically accompanied with a free-tissue reconstruction and with unilateral or bilateral modified radical neck dissection. Reconstruction after a total laryngectomy was performed by primary closure, with myocutaneous flap reconstruction, or with free tissue transfer. Adjuvant therapy after surgery consisted of concurrent radiotherapy or chemoradiotherapy using computed tomography-planned intensity-modulated radiotherapy with photons from a megavoltage linear accelerator. Hypopharyngeal and certain laryngeal and oropharyngeal tumors were treated with definitive chemoradiotherapy. Radiotherapy was given once a day and all patients received standard 60–70 Gy in 30–35 fractions over a period of 6–7 weeks. Treatment fields included the primary tumor site and neck. All patients received standard cisplatin-based regimens as adjuvant radiosensitisers.

Adverse events of chemoradiotherapy were classified according to the National Cancer Institute Common Terminology Criteria for Adverse Events-3.0 (CTCAEv3.0) (22). Assessment was performed before initiation of chemoradiotherapy, at the second week and at the end of treatment. Objective grading was performed by a radiation oncologist (KS) or by a dietician (HO) and Grade 3 adverse events were classified as severe adverse events.

### Nutritional Intervention

At our institution prophylactic PEG is inserted to almost all HNSCC patients either prior to surgery or before the start of chemoradiotherapy. Tube feeding was planned and instructed (i.e., enteral formula, volume [ml/day]) for all 51 patients with PEG according to personalized energy and protein requirements. Nutritional requirements were calculated according to WHO formula multiplied by activity factor 1.5 (23) and protein 1.3–1.5 g/kg/day (BMI 22). In this study, PEG was inserted for 88% of patients. Three patients referred for chemoradiotherapy did not consent to PEG placement, while 4 patients were considered not to need PEG. Nutritional counseling in both study groups was individualized given by a dietician, aiming to achieve and maintain patient's calculated energy and protein intake during (chemo) radiotherapy, either by PEG or via oral route.

Enteral formulas containing either 1 kcal/ml (0.3 g/ml protein, Nutrison® Multi Fiber, Nutricia, N.V. Nutricia, Zoetermeer, Holland) or 1.5 kcal/ml energy (0.6 g/ml protein, Nutrison® Energy Multi Fiber) were used. The INC group patients started tube feeding according to dietitian's instructions during RT. The ODC group patients were instructed to start tube feeding when their dietary intake was <60% of the usual intake or at the latest on the third week of RT.

All patients received protocoled nutritional counseling, which included dietary prescription (i.e., tube feeding, product, and volume) and counseling for energy- and protein-dense texture-modified diet via a dietary booklet. The latter booklet included meal plans and recipes for energy- and protein-dense texture modified meals for oral intake designed for the current study (15 pages). During chemoradiotherapy the INC group included a protocol assessment of nutritional intake and fine-tuning nutrition plan or nutrition for tube feeding according to treatment side-effects. ODC was done when requested by the attending physician.

### Nutritional Status

Patient characteristics included: age, sex, body weight (kg), height (cm), and recalled body weight at 1 and 6 months, previous to surgery, to definitive chemoradiotherapy or radiotherapy alone; hereafter named as chemoradiotherapy (baseline). Body weight, handgrip strength, and the full version of the PG-SGA (Data Sheet 1) were evaluated at baseline and at the sixth or seventh week of chemoradiotherapy (end). Dietary intake related problems were evaluated by PG-SGA (24). Patients were classified as well-nourished (PG-SGA A) or malnourished (PG-SGA BC), and a total PG-SGA score was calculated as described by Ottery (24, 25). Permission for the full form of scored PG-SGA was received from Pt-Global (http://pt-global.org/).

### Weight and Body Composition

All patients were weighed using the same calibrated scale (Tanita, Illinois, USA®). The criterion for pre-treatment critical weight loss was either ≥5% in 1 month or ≥10% in the previous 6 months (11, 26–28). Critical weight loss during treatment was defined as >5% unintentional weight loss of baseline weight (29) and it was categorized into 2 groups: ≤5, >5%. BMI was classified as underweight if < 20 kg/m^2^, normal 20–25 kg/m^2^, or overweight if >25 kg/m^2^ (30).

Body composition was assessed by bioimpedance using a single frequency (50 kHz) two-terminal bio-impedance meter (Bodystat Ltd, Isle of Man, UK®) performed according to standard procedure (31), by the same dietician (HO). Patients were in supine position with no body parts touching the torso. Electrodes were placed on the patient's right hand and foot using a four-surface standard electrode technique.

Total body water was calculated by the Kotler equations (32). The theoretical normal hydration of fat-free tissue was assumed and calculated as 0.73. Fat mass (FM) was calculated as the difference between body mass and fat-free mass (FFM). Percentage of FM was calculated by dividing total weight of FM (kg) by total body weight (kg) and FFM percentage subtracting FM percentage from 100. Fat-free mass index (FFMI) was calculated by dividing FFM (kg) by the square of the body height (m^2^).

### Muscle Mass Function

Skeletal muscle function was assessed by measuring HGS for both arms with a JAMAR® dynamometer (Sammons Preston Rolyan, Chicago, USA®). Patients performed the test while sitting comfortably with adducted shoulders and forearm neutrally rotated, elbow flexed to 90°, and forearm and wrist in neutral position. According to the manufacturer's instructions, the instrument was adjusted for hand size; position “3” was used for men, and position “2” for women. Patients were instructed to perform a maximal isometric contraction. The test was repeated within 30 s and the mean of three measurements for the dominant hand was used for the analysis (33, 34). Values were compared with age and sex appropriate reference values. A cut-off point below the 5th percentile was used as an indicator of muscle mass dysfunction (34).

### Survival

Overall survival (OS) was defined as the time interval between the date of the randomization and the date of the last visit or death by any cause. Disease-specific survival (DSS) was defined as percentage of patients who had not died from HNSCC during that time period. The assessment period was from the time of diagnosis to the date of last visit or HNSCC-related death. Death from other cause than HNSCC was not defined as an event for DSS. Disease-free survival (DFS) was calculated from the completion of treatment to the detection of cancer recurrence or death of any cause according to the National Cancer Institute (http://www.cancer.gov/dictionary?cdrid=44023). The follow-up of HNSCC patients at our institution is scheduled according to the national protocol and therefore, there was no difference between the study groups regarding this issue.

### Sample Size Calculation

We performed power analysis before the beginning of the study. A sample size of 102 patients was identified to achieve 30% reduction in prevalence of malnutrition at the end of treatment (50–20%), with a significance value of 5% (*p* < 0.05) and 90% power. The required number of patients decreased to 88 when we updated the calculation later and decreased the power requirement from 90 to 85%. Based on the updated calculations, our aim was to recruit 100 patients, with the assumption that 12% of patients would be lost to follow-up.

However, during 18 months of recruitment we managed to recruit only 65 patients due to many recruitment problems and it was clear that the number of 100 patients was unrealistic to be achieved. With the sample size of 65 patients (26 in INC and 32 in ODC), the estimated power was only 60% to show the difference (50–20%) statistically significant. Although an interim analysis was not preplanned in the study protocol, the check of apparent treatment difference with respect to the primary outcome measure, nutritional status, was needed. In the interim analysis of 58 patients, 32 patients in the INC group, and 26 patients in the ODC group, the baseline-adjusted odds ratio for malnutrition was 1.70 (95% CI 0.43–6.79, *p* = 0.45) when ODC was compared to INC. Although the treatment difference was not statistically significant, the study was discontinued due to appearance of a negative trend, where the direction was opposite to the one expected and because the 95% confidence interval did not include the predefined relative risk reduction of 60%. With a half-sample-size, INC superior to ODC (even if not significant) makes it very unlikely to obtain a significant opposite result with the planned sample size. The conditional power i.e., probability of obtaining a significant relative risk reduction considering the intermediate results is <58%.

### Statistical Analysis

For statistical data analyses, we included patients who fulfilled the treatment plan with follow-up appointments and assessments and did not perform an intention-to-treat analysis. All statistical analyses were performed with SPSS Statistical software (Version 19.0, IBM Corp., Armonk, NY, USA®).

Logistic regression analysis was used in the interim analysis when the presence of malnutrition (based on PG-SGA) in ODC was compared to INC. The baseline nutrition status was included as a categorical covariate. The result is given as odds ratio (OR) with 95% confidence interval. Weight, BMI, FFM, FFMI, FM, and HGS were normally distributed for both INC and ODC groups, as assessed by Shapiro-Wilk's test (*p* > 0.05). The homogeneity of regression slopes was attained and standardized residuals for the interventions were normally distributed, as assessed by Shapiro-Wilk's test (*p* > 0.05). There was homoscedasticity, as assessed by visual inspection of the standardized residuals plotted against the predicted values. There was homogeneity of variances, as assessed by Levene's test of homogeneity of variance. There were no outliers in the data, as assessed by no cases with standardized residuals > ±3 standard deviations. Descriptive data were expressed as median (interquartile ranges, IQ range).

Between-group comparisons were analyzed with one-way ANCOVA or two-way ANCOVA when appropriate. Between-group comparisons were analyzed with non-parametric Mann-Whitney's *U*-test or Kruskal-Wallis tests, as appropriate. All patients were kept in the group (INC *n* = 26, ODC *n* = 32) where they were assigned by the randomization system. Repeated measures were analyzed by Sign test or Wilcoxon signed-ranks test, and categorical variables by related samples McNemar test; category variables. Prevalence or frequency were evaluated by the *X*^2^ test. Correlations were analyzed by non-parametric Spearman rho and Kendal's tau. Prevalence and frequency were expressed as number and percentage. The Kaplan–Meier method was used to calculate survival and the log-rank test statistic was used to evaluate the equality of survival distributions across different strata. A two-tailed *p*-value below 0.05 was considered statistically significant.

## Results

### Patients

**Figure 1:**
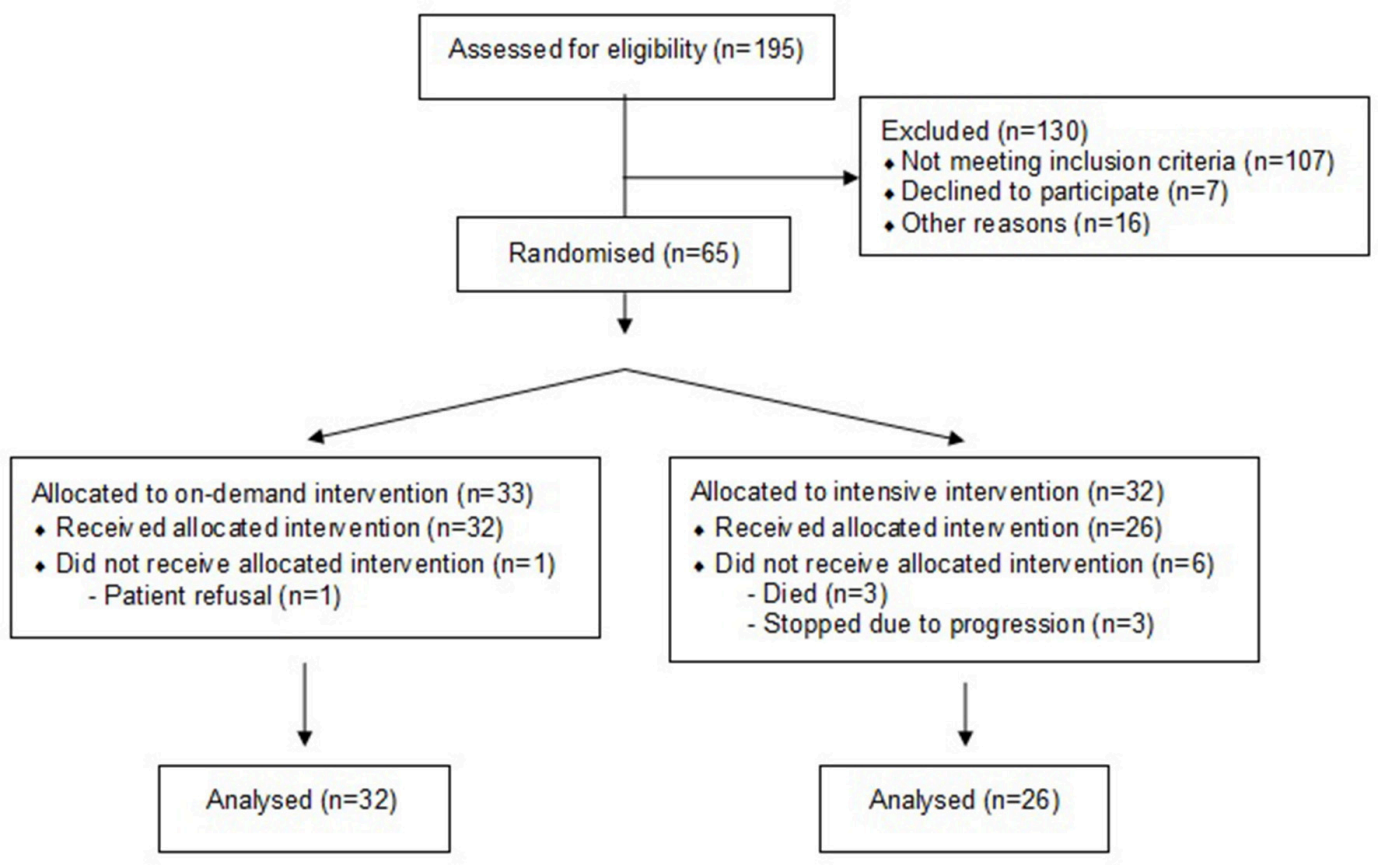
Study flowchart.

Altogether, 88 patients were eligible for inclusion and 65 (74%) gave their written informed consent to participate in the study. There were 23 patients who were not included in the study, 7 patients denied participation, 2 participated in another clinical trial, and 14 were lost due to logistic reasons (Figure 1). Of the 65 participants, 32 were randomly assigned to receive intensive individualized counseling (INC) and 33 to receive individualized on-demand counseling (ODC). Fifty-eight patients completed the treatment assessment, 1 patient withdrew his consent, 3 died and 3 discontinued their treatment due to disease progression. Most patients were male (79%), 22% were >65 years old, and the median age (range) for the 58 study patients, was 61 years old (33–73) for men and 59 years old (42–73) for women. The minimum follow-up time for both groups (INC and ODC) was 40 months or until death.

With the recruitment of the 65 patients, we maintained a significance value of 5% (*p* < 0.05) with 60% power. At this time point, and with 58 patients recruited for 2 years, we had to perform an unplanned pre-analysis due to recruitment problems. In the interim analysis of 58 patients, 32 patients in the control group (ODC) and 26 patients in the treatment group (INC), the baseline-adjusted odds ratio for malnutrition (PG-SGA BC) was 1.70 (95% CI 0.43–6.79, *p* = 0.45).

Baseline characteristics of the two study groups (INC and ODC) are presented in Tables 1, 2. The two groups were comparable and homogeneous at baseline in terms of age, sex, tumor location and stage. In the ODC group there were significantly more patients with T4 tumor stage (*p* = 0.02), and PEG dependence (*p* = 0.002), yet groups were balanced for PEG; the ODC group more often received chemoradiotherapy (*p* = 0.03) vs. patients in the INC group. Likewise, baseline variables did not differ between groups. The ODC group had more patients with malnutrition (*p* = 0.03), prophylactic PEG (*p* = 0.001), and chemoradiotherapy (*p* = 0.03) compared with those in the INC group.

**Table 1:** Baseline characteristics of the two study groups.

**Baseline characteristics**	**INC**	**ODC**	***p*-value**^**a**^
**Number of patients (%)**	26 (45)	32 (55)	
**Age y, median (IQ range)**	57 (52–64)	61 (56–64)	0.320
**≥65 y**, ***n*** **(%)**	6 (23)	7 (22)	0.365
**Males**, ***n*** **(%)**	21 (81)	25 (78)	0.412
**BMI, kg/m**^**2**^, ***n*** **(%)**			
<20	3 (12)	6 (19)	0.095
20–25	9 (35)	15 (47)	0.089
>25	14 (54)	11 (34)	0.869
**Malnourished (PG-SGA BC)**, ***n*** **(%)**	7 (27)	14 (44)	**0.039**
**PEG**, ***n*** **(%)**	20 (78)	31 (97)	**0.002**
**Tumor location**, ***n*** **(%)**			
Oral cavity	3 (12)	3 (9)	0.363
Oropharynx	11 (43)	13 (41)	0.399
Hypopharynx	4 (15)	5 (16)	0.292
Larynx	5 (19)	7 (22)	0.236
Nasopharynx	2 (8)	4 (12)	0.106
Unknown	1 (4)	–	
**Stage**, ***n*** **(%)**			
I	1 (4)	1(3)	0.190
II	2 (8)	4 (12)	0.106
III	6 (23)	6 (19)	0.475
IV	16 (62)	21 (66)	0.237
Unknown	1 (4)	–	
**Stage III–IV**, ***n*** **(%)**	22 (85)	27 (84)	0.309
**T Class**, ***n*** **(%)**			
T1	7 (27)	4 (12)	0.816
T2	7 (27)	8 (25)	0.395
T3	6 (23)	8 (25)	0.269
T4	5 (19)	12 (38)	**0.022**
Unknown	1 (4)	–	
**N Class**, ***n*** **(%)**			
N0	9 (35)	11(34)	0.352
N1	3 (12)	3 (9)	0.363
N2	13 (50)	18 (56)	0.196
N3	1 (4)	–	
**Treatment plan**, ***n*** **(%)**			
Surgery + chemoradiotherapy	6 (23)	5 (16)	0.593
Surgery + radiotherapy	1 (4)	1 (3)	0.190
Chemoradiotherapy	16 (61)	25 (78)	**0.036**
Radiotherapy	3 (12)	1 (3)	0.688
**Smoking, pack years**, ***n*** **(%)**	17.5 (1–36)	37.6 (12–54)	**0.013**
**Alcohol, drinks per week**, ***n*** **(%)**	2.5 (1–9)	9 (1–21)	0.088

*IQ interquartile, BMI body mass index, PG-SGA, patient generated subjective global assessment; T, tumor; N, node; PEG, percutaneous gastrostomy; INC, intensive nutrition counseling; ODC, on-demand nutritional counseling. Data expressed as median (interquartile range) or number (%)*.

a *p-value Mann-Whitney or X^2^ test between INC and ODC group*.

**Table 2:** Nutritional characteristics at baseline and end of treatment for all 58 patients and for two study groups.

	**INC** **(*n* = 26)**		**ODC** **(*n* = 32)**		**Baseline adjusted mean difference** **at the end of treatment, Mean (95% CI)**^**a**^		
**Characteristics**	**Baseline**	**End**	**Baseline**	**End**	**INC**	**ODC**	***P*-value**
Weight, kg	79.2 (63–87)	71.4 (58–81)	69.7 (57–82)	65.8 (55–78)	68.3 (67.0, 69.6)	68.7 (67.5, 69.8)	0.690
BMI, kg/m^2^	24.8 (22–28)	23.3 (20–26)	23.1 (21–26)	22.4 (19–24)	22.7 (22.2, 23.1)	22.8 (22.4, 23.1)	0.656
FFMI, kg/m^2^	19.4 (18–22)	18.8 (16–20)	18.8 (27–20)	17.4 (16–20)	18.3 (17.8, 18.7)	18.4 (18.0, 18.7)	0.741
FFM, kg	62.0 (48–69)	57.0 (47–64)	58.8 (48–62)	54.0 (46–62)	55.1 (53.8, 56.4)	55.4 (54.2, 56.6)	0.684
FM, kg	16.9 (10–21)	14.0 (10–17)	14.0 (10–20)	11.1 (10–16)	13.4 (12.6, 14.2)	12.8 (12.1, 13.6)	0.332
HGS, kg	37.4 (31–49)	30.7 (26–46)	36.4 (26–43)	31.4 (23–40)	33.8 (32.1. 35.5)	33.5 (31.9, 35.1)	0.803

Data presented as median, IQ (interquartile) range. INC, intensive nutritional counseling; ODC, on-demand counseling; CI, confidence interval; BMI, body mass index; FFMI, fat-free mass index; FFM, fat-free mass; FM, fat mass; HGS, handgrip strength;

a *Baseline-adjusted mean difference (95% CI) at the end of treatment, where the baseline measurement is included as a continuous covariate (ANCOVA). INC group patients are compared to ODC group patients*.

Although the treatment difference was not statistically significant, the study was discontinued due to apparent negative trend, where the direction was opposite to the one expected and because the 95% confidence interval did not include the predefined relative risk reduction of 60%. With a half-sample-size as the one calculated and a control superior to active treatment (even if not significant) makes it unlikely to obtain a significant opposite result with the planned sample size. The conditional power i.e., probability of obtaining a significant relative risk reduction considering the intermediate results is <58%.

### Nutritional Outcomes

At the end of treatment, overall parameters were worse and there were no significant differences between study groups (Table 2). In detail concerning the primary endpoint, the prevalence of malnutrition according to PG-SGA increased during treatment from 27 to 85% in the INC group (*p* < 0.001) and from 44 to 75% in the ODC group (*p* = 0.007). Median PG-SGA score for all patients increased significantly during treatment (*p* < 0.001). At baseline, 22% (*n* = 13/58) of patients needed critical intervention (≥9 points), and at the end of treatment this proportion of patients increased to 71% (*n* = 41/58). There was no difference in body composition and function between the two study groups neither at baseline nor at the end of treatment (Table 2).

### Weight Loss

At baseline, critical weight loss was seen in 24% (*n* = 14/58) of all patients. Ten out of these 14 patients had this weight loss during the previous month (median 6.1%, IQ range 5–10) and 11 patients reported critical weight loss in the previous 6 months (12.1%, 12–14). Fifty percent (*n* = 7/14) had critical weight loss both during the previous month and in the past 6-month period. Critical pre-treatment weight loss was similar in the two study groups: 23% (*n* = 6/26) in the INC and 25% (*n* = 8/32) in the ODC (*p* = 0.269). In a similar fashion, at the end of chemoradiotherapy, 71% (*n* = 41/58) of all patients had lost >5% of weight with a median weight loss of 7.7% (IQ range 7–11). Critical weight loss was similar in both groups: 77% (*n* = 20/26) in the INC group (median 8.4%, IQ range 7–11) and 67% (*n* = 21/32) in the ODC group (median 7.1%, IQ range 7–10, *p* = 0.704).

An additional finding was that prevalence of overweight patients decreased from 43 to 26% (*p* = 0.921), and underweight patients increased from 16 to 28% (*p* = 0.012). Forty-one percent of patients in the INC group and 47% in the ODC group remained within normal weight range, and there were no significant differences between groups.

### Nutritional Intake

At the end of treatment, overall median (IQ range) for energy intake was 82% (68–91) of estimated requirements and median protein intake was 72% (60–81) of estimated requirements; we did not find significant differences between the two study groups. In detail, median energy intake was 27.5 kcal/kg in the INC group (2,000 kcal/day) and 29.5 kcal/kg (1,950 kcal/day) in the ODC group (*p* = 0.24, NS). Regarding energy intake, 26% of all patients reached >90% of estimated energy needs, 19% (*n* = 5/26) of them in the INC and 31% (*n* = 10/32) in the ODC group (INC vs. ODC, *p* = 0.06). Furthermore, 12% of all patients reached >90% of estimated protein needs: three patients in INC and four in the ODC group, *p* = 0.243).

Six (10%) of all patients were PEG dependent prior to diagnosis, while 45 patients started to use PEG on median (IQ range) of 16th (11–22) day from the start of chemoradiotherapy. The median (IQ range) PEG dependency was 4 (2–9) months. One patient in the ODC group denied using PEG. There was no difference in the follow-up regarding PEG dependence and usage as a nutritional route.

### Handgrip Strength

At baseline, median (IQ range) HGS in females was 22 kg (20–31) and 39 kg (31–49) for men; at the end of treatment, results were 22 kg (18–29) and 35 kg (28–46), respectively. There were no significant differences between INC and ODC groups. At baseline malnourished patients (PG-SGA BC) had significantly lower HGS (*p* = 0.001) than well-nourished patients (PG-SGA A) and it decreased during treatment in both sub-groups. Median (IQ range) HGS in malnourished patients was at baseline 28 kg (22–38) and at the end of treatment 27 kg (20–31) (*p* = 0.24), in well-nourished patients 39 kg (33–49) and 38 kg (29–47) (*p* = 0.001), respectively. Low HGS at baseline (<5th percentile reference values) was seen in 17% (*n* = 10/58) and at the end of treatment in 31% (*n* = 18/58) of all patients (*p* = 0.008).

### Treatment-Related Adverse Events

Sixty-one per cent (*n* = 14/23) of patients in the INC group completed their prescribed chemotherapy and 60% (*n* = 18/30) in the ODC group (*p* = 0.326); 92% (*n* = 24/26); and 91% (*n* = 29/32) completed their scheduled radiotherapy, respectively (*p* = 0.380). In both groups, five patients were admitted to the hospital during chemoradiotherapy and one patient had to discontinue radiotherapy for >5 days in the INC group. Severe mucositis was seen in 19% (*n* = 5/26) of patients in the INC group and in 25% (8/32) of patients in the ODC group (*p* = 0.161).

Of the patients who had lost ≤10% of weight during treatment, 48% (*n* = 21/44) completed the planned chemotherapy, 89% (*n* = 39/44) completed their scheduled radiotherapy, and 23% (*n* = 10/44) had hospital admissions. In contrast, for patients with >10% weight loss these figures were 86% (*n* = 12/14) and 100% (*n* = 14/14), respectively (*p* < 0.001), and they had no hospital admissions. Severe mucositis was seen in 25% (*n* = 11/44) patients with ≤10% weight loss vs. 14% (*n* = 2/14) of patients with weight loss >10% (*p* = 692). Severe nausea was seen significantly more in patients with >10% weight loss as compared with patients with ≤10% weight loss, 29% (*n* = 4/14) vs. 5% (*n* = 2/44), *p* = 0.01. A linear correlation was found between percentage weight during treatment and the severity of anorexia in all patients (*r* = −0.34; *p* < 0.01) explaining the 11% variation in weight loss.

Majority of all patients (69%, *n* = 40/58) were not able to carry out their planned nutritional treatment. The main patient-reported problems for unsuccessful nutritional intake were nausea (22%, *n* = 13/58), early satiety (12%, *n* = 7/58), loss of motivation (9%, *n* = 5/58), or miscellaneous reasons (21%, *n* = 12/58, e.g. PEG related causes, exhaustion, cachexia, financial issues and severe diarrhea).

### Survival

Median follow-up for all study participants was 43 months (range 6–63); 46 months in the INC group and 42 months in the ODC group (*p* = 0.189). The 5-year OS for all patients was 60; 69% (18/26) in the INC group and 53% (17/32) in the ODC group (*p* = 0.81). DSS rates were 75% (18/24) and 68% (17/25), respectively (*p* = 562), and DFS rates 65% (17/26) and 41% (13/32), respectively (*p* = 939). DSS and DFS for all patients were 71 and 52%, respectively.

**Figure 2:**
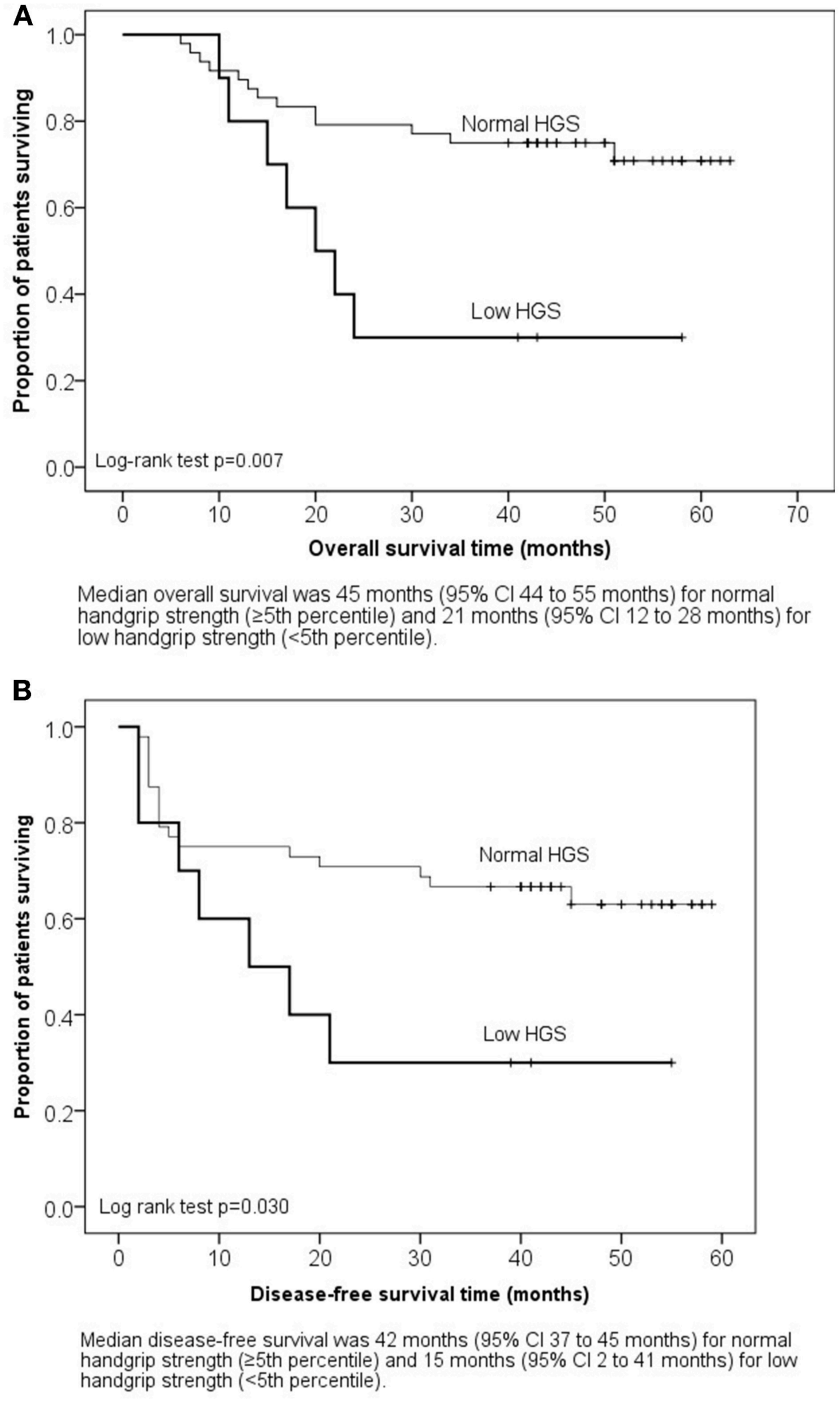
**(A)** Kaplan-Meier survival plot: overall survival by baseline handgrip strength. **(B)** Kaplan-Meier survival plot: disease-free survival by baseline handgrip strength.

The 5-year OS for patients with baseline low HGS was 20% (2/10) and for normal HGS 69% (33/48, *p* = 0.004). The DFS rates were 20% (2/10) and 58% (28/48), respectively (*p* = 0.03). Kaplan-Meier survival analysis showed that patients with low HGS (< 5th percentile) at baseline had significantly poorer median OS (Figure 2A) and shorter DFS (Figure 2B) than patients with normal HGS. The 5-year OS for patients with baseline malnutrition (PG-SGA BC) was 43% (9/21) and for well-nourished 70% (26/37, *p* = 0.04). The median OS was 38 months (95% CI 28–47 months) for malnourished patients and 50 months (95% CI 43–56 months) for well-nourished patients (Log-rank test *p* = 0.035).

HNSCC was the primary cause of death in 22% (*n* = 13/58) of all patients; local recurrence was seen in 12 patients and distant metastases in one patient in INC group. In the INC group six patients died due to HNSCC and one due to pneumonia. In the ODC group seven died due to HNSCC and the other causes of death were secondary cancers (*n* = 2), ileus, pneumonia (*n* = 2), and unknown cause of death (*n* = 2).

### Efficacy of Nutritional Intervention

Proportional ratios of patients that maintained good nutritional status (PG-SGA A), good FFM (FFM loss < 5%), normal HGS (>5th percentile) and stable weight (weight loss ≤ 5%) in the INC and ODC groups were calculated to analyse the efficiency of nutritional intervention. In the INC group, 15% maintained a good nutritional status, 23% their body weight, 54% of patients maintained their muscle mass, and 77% their HGS, whilst in the ODC group, the respective proportions were 22, 32, 53, 66% (NS).

Nutritional characteristics according to baseline nutritional status and BMI are shown in Table 3. There was a trend for higher pre-treatment BMI (≥25) to be associated with more severe weight loss compared with normal weight patients (BMI <25): median (IQ range) weight loss of 3.2 (1–5) kg vs. 7.1 (5–9) kg (*p* < 0.001), respectively. After adjustment for pre-intervention weight loss, there was a statistically significant difference in treatment induced weight loss between two BMI groups, *F*_(1, 51)_ = 5.609, *p* = 0.021, partial η^2^ = 0.093 and baseline nutritional status groups, *F*_(1, 55)_ = 5.580, *p* = 0.022, partial η^2^ = 0.093 (Table 3). *Post-hoc* analysis was performed with a Bonferroni adjustment. Treatment-induced weight loss was statistically significantly greater in the group with baseline overweight (BMI ≥ 25 kg/m^2^) vs. the group with normal weight (BMI < 25 kg/m^2^) with the mean difference of 2.8 (95% CI, 1.127–4.373) kg*, p* < 0.001. Despite weight loss, malnourished patients did not lose FFM during treatment (*p* = 0.033). At the end of treatment overweight patients and well-nourished patients still had significantly higher FFM (*p* = 0.005 and *p* < 0.001, respectively) and HGS (*p* = 0.001 and *p* < 0.001, respectively) despite weight loss. Malnourished patients had higher median (IQ range) energy intake 31 (28–32) kcal/kg) at the end of treatment as compared with the well-nourished patients [26 (21–20) kcal/kg, *p* = 0.010]. The same trend was seen also between those with normal weight and overweight patients at baseline; 31 (28–34) kcal/kg, and 25 (21–28) kcal/kg, respectively (*p* < 0.001).

**Table 3:** Nutritional characteristics in patients according to baseline nutritional status and BMI.

	**Baseline nutritional status (PG-SGA)**		**Baseline BMI, kg/m**^****2****^	
Characteristics	Well-nourished	Malnourished	< 25	≥25
Number of patients, (%)	37 (64)	21 (36)	33 (57)	25 (43)
**Weight loss, kg**				
6 mo pre-treatment	0.6 (^−^2 – 1)	6.3 (^−^6 – ^−^1)	4.5 (0.1–7)	0.7 (0–4)
During treatment	5.5 (^−^11 – ^−^5)	3.2 (^−^8–^−^1)	3.2 (1–5)	7.1 (5–9)
Difference^a^	5.1 (4.0, 6.2)	4.3 (2.7, 5.0)	3.5 (4.9, 2.1)	6.6 (8.2, 4.9)
*p*-value		0.476		**0.021**
**Weight**				
Baseline	79.7 (68–89)	62.9 (53–76)	63.8 (56–68)	86.1 (82–93)
End	73.3 (62–81)	58.9 (53–70)	58.2 (53–66)	80.1 (74.86)
Difference^a^	68.0 (67.0, 69.1)	69.3 (67.9, 70.8)	69.0 (67.6, 70.4)	67.8 (66.1, 69.6)
*p*-value		0.158		0.368
**BMI**				
Baseline	24.8 (23–28)	21.8 (18–25)	21.6 (19–23)	27.4 (26–29)
End	23.6 (21–26)	20.3 (19.23)	25.2 (24–26)	25.2 (24–26)
Difference^a^	22.6 (22.2, 22.9)	23.0 (22.6, 23.5)	23.0 (22.5, 23.4)	22.5 (21.9, 23.0)
*p*-value		0.144		0.269
**FFM, kg**				
Baseline	61.3 (54–68)	47.2 (42–61)	51.1 (44–58)	66 (61–73)
End	58.6 (50–64)	47.4 (42–56)	47.0 (43–55)	64 (57–70)
Difference^a^	55.0 (53.9, 56.1)	55.7 (54.1, 57.2)	18.1 (17.6, 18.5)	18.6 (18.1, 19.1)
*p*-value		0.459		0.202
**FFMI, kg/m**^**2**^				
Baseline	19.9 (19–22)	16.9 (14–20)	16.9 (16–19)	21.0 (20–23)
End	19.3 (17–20)	16.3 (16–18)	16.2 (16–18)	20.1 (19–22)
Difference^a^	18.3 (17.9, 18.6)	18.4 (17.9, 18.9)	18.1 (17.6, 18.5)	18.6 (18.0, 19.2)
*p*-value		0.249		0.202
**HGS, kg**				
Baseline	39.3 (33–49)	28.0 (22–37)	31.3 (22–37)	42.3 (38–53)
End	38.0 (29–47)	27.3 (20–31)	28.0 (21–36)	40.7 (31–49)
Difference^a^	34.2 (32.8, 35.7)	32.6 (30.6, 34.6)	33.4 (31.7, 35.1)	32.0 (32.0, 35.9)
*p*-value		0.214		0.705

*Data presented as median, IQ (interquartile) range. FFM, fat-free mass; FFMI, fat-free mass index; HGS, handgrip strength; PG-SGA, patient generated subjective global assessment; OS, overall survival; DFS, disease-free survival*.

a *Baseline-adjusted mean difference (95% CI) at the end of treatment, where the baseline measurement is included as a continuous covariate (ANCOVA). Well-nourished patients are compared to malnourished patients and patients with BMI < 25 kg/m^2^ are compared to patients with BMI ≥ 25 kg/m^2^*.

There was no statistically significant correlation between type of nutritional counseling (INC vs. ODC) and baseline nutritional status (according to PG-SGA) for weight, weight loss, FFM, FFMI, FM, and HGS analyzed by two-way ANCOVA (Data not shown).

## Discussion

The aim of the study was to evaluate the effect of nutritional intervention on HNSCC patients' nutritional status in a prospective randomized trial setting with two modalities. Aware of the limitations of this study inherent from the reduced number of patients in each study arm, we can only provide exploratory data. However, by paired analyses between the studied groups, we detected a trend for intensive nutrition counseling and on-demand counseling to stabilize nutrition status equally in patients with pre-treatment weight loss. Furthermore, these were not able to prevent FFM loss in these populations except in baseline malnourished patients.

There were no differences in the two nutritional counseling methods on nutritional status, or in any of the secondary endpoints (FFM, HGS, survival). Pre-treatment low HGS and malnutrition by PG-SGA were associated with poorer survival whereas treatment induced weight loss or malnutrition did not. This might be due to the research protocol used in this study. We did not compare results with a control group without any nutrition; instead we compared two modalities of nutritional intervention during treatment. This study also showed that physicians and nurses paid attention to nutritional problems and used dietician services appropriately for the on-demand group. We can almost presume that this study explored whether there was an important signal to indicate a difference between the two treatment groups in an exploratory randomized trial to further explore nutritional and clinical endpoints in a larger randomized controlled trial.

Previous studies have reported that nutritional counseling has a positive influence on nutritional intake and clinical outcomes in patients with HNSCC undergoing radiotherapy (2, 13, 14, 16) or chemoradiotherapy (5, 6). Four studies showed that nutritional intervention given by a dietician was more efficacious vs. no counseling or general nutritional advice given by a nurse (2, 13, 14, 16). Two studies conducted in patients with HNSCC receiving chemoradiotherapy have showed that weight loss decreased in patients receiving nutritional intervention compared with no intervention, while compliant patients lost less weight than non-compliant patients (5, 6); it has also been suggested that nutrition may improve long-term outcomes in colorectal cancer (20).

Nutritional counseling seemed more efficacious in patients with more profound pre-treatment weight loss as these patients lost less weight during their treatment compared with those with no pre-treatment weight loss. This is contrary to previous data showing that weight loss tends to persist during oncologic treatment (6). The current study additionally showed that overweight patients (BMI > 25 kg/m^2^) lost significantly more weight due to more profound treatment-induced anorexia and nausea. They still had clinically better muscle strength and muscle mass compared with patients with BMI ≤ 25 at the end of treatment. This might explain the finding that treatment induced weight loss was not associated with survival. Instead, patients with either pre-treatment low HGS (< 5th percentile) or malnutrition (PG-SGA BC) had shorter DFS compared with baseline normal HGS and good nutritional status.

Despite the fact that we did not find differences between nutritional intervention groups, from a clinical point of view it is important to refer that 69% of patients had difficulties in achieving the planned nutritional treatment, which is in line with the study by Capuano (53%) (6). The possible cause might be the (chemo) radiotherapy induced adverse events, such as nausea and anorexia, which were seen more often in overweight patients. As the majority of patients were obese, this had impact on study results and might require more aggressive treatment of therapy related adverse events in the future.

Malnutrition before treatment has been found in 60% of patients with HNSCC according to PG-SGA, and at the end of chemoradiotherapy it may increase up to 85% (2, 5). In line with previous studies, we found malnutrition by PG-SGA to increase from 31 to 86%. It is noteworthy that in PG-SGA, a weight-losing patient is classified as malnourished even if the muscle mass loss is substantial but still over 50th percentile. Our results indicate that even though overweight patients lost significantly weight they had at the end of chemoradiotherapy higher muscle mass and HGS in comparison with low or normal BMI. Moreover, patients with pre-treatment malnutrition had shorter survival and DFS in comparison with well-nourished patients.

Weight loss is a dominant feature among HNSCC patients. Previous studies have reported a median weight loss before chemoradiotherapy to be 4% and at the end of treatment to reach 10% (6), which is in accordance with our results (7–8%). Weight loss of more than 20% during chemoradiotherapy has been associated with impaired survival (6, 17). In the current study weight loss was not associated with impaired survival due to that none of the patients lost weight more than 15% and the median weight loss for all patients was < 10%. Instead, we found that overweight patients had higher risk of severe weight loss, which has been observed previously among cancer patients in general, as well as among Stage III-IV HNSCC patients (35, 36). It seems that patients with higher pre-treatment BMI had more nausea and anorexia, and thus greater weight loss. This was also seen in the study by Prado et al. (37), which showed that patients with sarcopenic obesity had more chemotherapy related adverse events. Furthermore, overweight patients may have more cytokines from adipose tissue delaying gastric emptying and inducing nausea and anorexia (38). On the other hand, energy requirements for overweight patients are higher and harder to achieve during chemoradiotherapy due to delayed gastric emptying, which may have led to impaired nutrition through PEG. One explanation could be that patients with lower BMI were after all heavy alcohol users, and thus had less chemotherapy related adverse events such as nausea, vomiting, and anorexia (39).

HGS has been shown to predict nutritional status and survival in advanced stage cancer patients (40, 41). In our study, malnourished patients had 29% lower HGS compared with well-nourished ones, which is in line with a previous study (40). Our results showed that patients with baseline HGS <5th percentile had markedly shorter survival and disease-free survival. Based on these results, the association between HGS <5th percentile and survival clearly needs to be validated in a larger cohort.

Energy and protein need should be at least 20% higher than in current study. Based on the fact that weight stable patients achieved 90% of the energy and protein intake while weight losing patients achieved only 70%. Furthermore, a tube feed with 2 kcal/ml of energy and 10 g/100 ml of protein might be more preferably than a product with 1.5 kcal/ml and 6 g/100 ml, which was used in the current study. The perceived anorexia and fullness was seen especially in obese patients, which needs more attention.

This exploratory prospective randomized trial has several strengths: all patients received either radiotherapy or chemoradiotherapy with curative intent for their primary HNSCC. Nutritional status was assessed with validated methods, performed by a single dietician. The number of patients was lower than intended and the low sample size may have led to Type II errors. Due to the small number of patients these results should be handled with caution. However, the present series comprised 74% of all eligible cases and was therefore considered to be representative for the HNSCC patient population at our institution. Yet, this study shows the difficulty of recruiting an adequate sample size and highlights the importance of conducting multi-center studies in the future. Furthermore, these data can be used for organizing and targeting nutritional therapies more appropriately and cost efficaciously by allocating high-risk patients (weight-losing patients) at the start and the format and duration of the therapy.

According to results of the current study we suggest that nutritional counseling given by a dietician should be prior to (chemo) radiotherapy and a follow-up visit at the third week of (chemo) radiotherapy when treatment related side-effects commonly appear. In addition, weight should be measured weekly by a nurse and a dietician referral on-demand if weight loss is seen. It is also clear that more effort should be devoted to the prevention of muscle mass loss.

To our knowledge, this is the first study comparing individualized intensive nutritional counseling with individualized on-demand nutritional counseling given by a dietician prior to and during oncologic treatment. On-demand nutritional counseling seemed not inferior to intensive counseling; this should be verified with larger number of patients. Regardless of weight loss, there was no significant difference in overall survival, completion of (chemo) radiotherapy or functional outcomes between the study groups. Worse HGS and malnutrition seemed to influence survival. The implementation of nutritional counseling adapted to the clinical context of each department seems mandatory. Furthermore, more attention should be given to overweight patients submitted to chemoradiotherapy. These results do emphasize the importance of establishing multimodal nutrition teams to efficaciously modulate individual nutritional aspects with the progession of the clinical status of such patients.

## Author Contributions

HO, AM, US, and KS designed the study. HO was the main author of the manuscript, performed all the assessments of nutritional status, analyzed, and interpreted data. AM supervised the project and assisted with the statistical analysis, writing the manuscript, and interpretation of the results. PÖ, PR, and US assisted in interpretation of the results and writing the manuscript.

### Conflict of Interest Statement

The authors declare that the research was conducted in the absence of any commercial or financial relationships that could be construed as a potential conflict of interest.
