# Supplementary material for: Nutritional Counseling for Head and Neck Cancer Patients Undergoing (Chemo) Radiotherapy—A Prospective Randomized Trial
Source: Front Nutr. 2019 Mar 18;6:22. doi: 10.3389/fnut.2019.00022 (PMC6432820; doi:10.3389/fnut.2019.00022)
Supplement: Supplementary file 1 [file Data_Sheet_1.PDF]

Scored Patient-Generated Subjective Global Assessment (PG-SGA)

History: Boxes 1 - 4 are designed to be completed by the patient.
[Boxes 1-4 are referred to as the PG-SGA Short Form (SF)]

Patient Identification Information

1. Weight *(See Worksheet 1)*

In summary of my current and recent weight:

I currently weigh about _____kg
I am about _____ cm tall

One month ago I weighed about _____ kg
Six months ago I weighed about _____ kg

During the past two weeks my weight has:

☐ decreased (1) ☐ not changed (0) ☐ increased (0)

Box 1 ☐

2. Food intake: As compared to my normal intake, I would rate my food intake during the past month as

- ☐ unchanged (0)
- ☐ more than usual (0)
- ☐ less than usual (1)

I am now taking

- ☐ *normal food* but less than normal amount (1)
- ☐ little solid food (2)
- ☐ only liquids (3)
- ☐ only nutritional supplements (3)
- ☐ very little of anything (4)
- ☐ only tube feedings or only nutrition by vein (0)

Box 2 ☐

3. Symptoms: I have had the following problems that have kept me from eating enough during the past two weeks (check all that apply)

- ☐ no problems eating (0)
- ☐ no appetite, just did not feel like eating (3)
- ☐ nausea (1)
- ☐ constipation (1)
- ☐ mouth sores (2)
- ☐ things taste funny or have no taste (1)
- ☐ problems swallowing (2)
- ☐ pain; where? (3) _______________
- ☐ other (1)** _________________
- ☐ vomiting (3)
- ☐ diarrhea (3)
- ☐ dry mouth (1)
- ☐ smells bother me (1)
- ☐ feel full quickly (1)
- ☐ fatigue (1)

Examples: depression, money, or dental problems **Box 3 ☐

4. Activities and Function:
Over the past month, I would generally rate my activity as:

- ☐ normal with no limitations (0)
- ☐ not my normal self, but able to be up and about with fairly normal activities (1)
- ☐ not feeling up to most things, but in bed or chair less than half the day (2)
- ☐ able to do little activity and spend most of the day in bed or chair (3)
- ☐ pretty much bed ridden, rarely out of bed (3)

Box 4 ☐

The remainder of this form is to be completed by your doctor, nurse, dietitian, or therapist. Thank you.

Additive Score of Boxes 1-4 ☐ **A**

©FD Ottery 2005, 2006, 2015 v3.22.15
email: faithotterymdphd@aol.com or info@pt-global.org

Scored Patient-Generated Subjective Global Assessment (PG-SGA)

Worksheet 1 – Scoring Weight Loss

To determine score, use 1-month weight data if available. Use 6-month data only if there is no 1-month weight data. Use points below to score weight change and add one extra point if patient has lost weight during the past 2 weeks. Enter total point score in Box 1 of PG-SGA.

Weight loss in 1 month	Points	Weight loss in 6 months
10% or greater	4	20% or greater
5-9.9%	3	10- 19.9%
3-4.9%	2	6- 9.9%
2-2.9%	1	2- 5.9%
0-1.9%	0	0- 1.9%

Numerical score from Worksheet 1 ☐

Additive Score of Boxes 1-4 (See Side 1) ☐ **A**

5. Worksheet 2 – Disease and its relation to nutritional requirements:

Score is derived by adding 1 point for each of the following conditions:

- ☐ Cancer
- ☐ Presence of decubitus, open wound or fistula
- ☐ AIDS
- ☐ Presence of trauma
- ☐ Pulmonary or cardiac cachexia
- ☐ Age greater than 65
- ☐ Chronic renal insufficiency

Other relevant diagnoses (specify) ______________________________

Primary disease staging (circle if known or appropriate) I II III IV Other ____

Numerical score from Worksheet 2 ☐ **B**

6. Worksheet 3 – Metabolic Demand

Score for metabolic stress is determined by a number of variables known to increase protein & caloric needs. **Note:** Score fever intensity or duration, whichever is greater. The score is additive so that a patient who has a fever of 38.8 °C (3 points) for < 72 hrs (1 point) and who is on 10 mg of prednisone chronically (2 points) would have an additive score for this section of 5 points.

Stress	none (0)	low (1)	moderate (2)	high (3)
**Fever**	no fever	> 37.2 and < 38.3	≥ 38.3 and < 38.8	≥ 38.8 °C
**Fever duration**	no fever	< 72 hours	72 hours	> 72 hours
**Corticosteroids**	no corticosteroids	low dose (< 10 mg prednisone equivalents/day)	moderate dose (≥ 10 and < 30 mg prednisone equivalents/day)	high dose (≥ 30 mg prednisone equivalents/day)

Numerical score from Worksheet 3 ☐ **C**

7. Worksheet 4 – Physical Exam

Exam includes a subjective evaluation of 3 aspects of body composition: fat, muscle, & fluid. Since this is subjective, each aspect of the exam is rated for degree. Muscle deficit/loss impacts point score more than fat deficit/loss. Definition of categories: 0 = no abnormality, 1+ = mild, 2+ = moderate, 3+ = severe. Rating in these categories is *not* additive but are used to clinically assess the degree of deficit (or presence of excess fluid).

Muscle Status				
temples (temporalis muscle)	0	1+	2+	3+
clavicles (pectoralis & deltoids)	0	1+	2+	3+
shoulders (deltoids)	0	1+	2+	3+
interosseous muscles	0	1+	2+	3+
scapula (latissimus dorsi, trapezius, deltoids)	0	1+	2+	3+
thigh (quadriceps)	0	1+	2+	3+
calf (gastrocnemius)	0	1+	2+	3+
**Global muscle status rating**	**0**	**1+**	**2+**	**3+**

Fat Stores				
orbital fat pads	0	1+	2+	3+
triceps skin fold	0	1+	2+	3+
fat overlying lower ribs	0	1+	2+	3+
**Global fat deficit rating**	**0**	**1+**	**2+**	**3+**

Fluid status				
ankle edema	0	1+	2+	3+
sacral edema	0	1+	2+	3+
ascites	0	1+	2+	3+
**Global fluid status rating**	**0**	**1+**	**2+**	**3+**

Point score for the physical exam is determined by the overall subjective rating of the total body deficit.

No deficit	score = 0 points
Mild deficit	score = 1 point
Moderate deficit	score = 2 points
Severe deficit	score = 3 points

Again, muscle deficit/loss takes precedence over fat loss or fluid excess.

Numerical Score for Worksheet 4 ☐ **D**

Total PG-SGA Score (Total numerical score of A+B+C+D) ☐

Clinician Signature ______________________________ RD RN PA MD DO Other ________ Date ____________

Global PG-SGA Category Rating (Stage A, Stage B or Stage C) ☐

Worksheet 5 – PG-SGA Global Assessment Categories

Category	Stage A<br>Well-nourished	Stage B<br>Moderate/suspected malnutrition	Stage C<br>Severely malnourished
**Weight**	No weight loss **OR** recent non-fluid wt gain	≤ 5% loss in 1 month (≤10% in 6 months) **OR** Progressive weight loss	> 5% loss in 1 month (>10% in 6 months) **OR** Progressive weight loss
**Nutrient intake**	No deficit **OR** Significant recent improvement	Definite decrease in intake	Severe deficit in intake
**Nutrition Impact Symptoms (NIS)**	None **OR** significant recent improvement allowing adequate intake	Presence of NIS (Box 3 of PG-SGA)	Presence of NIS (Box 3 of PG-SGA)
**Functioning**	No deficit **OR** Significant recent improvement	Moderate functional deficit **OR** Recent deterioration	Severe functional deficit **OR** Recent significant deterioration
**Physical Exam**	No deficit **OR** chronic deficit but with recent clinical improvement	Evidence of mild to moderate loss of muscle mass &/or muscle tone on palpation &/or loss of SQ fat	Obvious signs of malnutrition (e.g., severe loss muscle, fat, possible edema)

Nutritional Triage Recommendations:

Additive score is used to define specific nutritional interventions including patient & family education, symptom management including pharmacologic intervention, and appropriate nutrient intervention (food, nutritional supplements, enteral, or parenteral triage).

First line nutrition intervention includes optimal symptom management.

Triage based on PG-SGA point score

- **0-1** No intervention required at this time. Re-assessment on routine and regular basis during treatment.
- **2-3** Patient & family education by dietitian, nurse, or other clinician with pharmacologic intervention as indicated by symptom survey (Box 3) and lab values as appropriate.
- **4-8** Requires intervention by dietitian, in conjunction with nurse or physician as indicated by symptoms (Box 3).
- **≥ 9** Indicates a critical need for improved symptom management and/or nutrient intervention options.

©FD Ottery 2005, 2006, 2015 v3.22.15

email: faithotterymdphd@aol.com or info@pt-global.org
